# Development of a Chemiluminescence Immunoassay for the Serological Diagnosis of Sheep and Bovine Brucellosis

**DOI:** 10.3390/microorganisms13092214

**Published:** 2025-09-22

**Authors:** Xin Yan, Mingze Chen, Yuning Liu, Mingjun Sun, Mengkun Huang, Jihui Jin, Jiaqi Li, Xiangxiang Sun, Mengda Liu, Haobo Zhang, Weixing Shao, Shufang Sun, Xiaoxu Fan, Wenlong Nan

**Affiliations:** 1 Laboratory of Zoonoses, China Animal Health and Epidemiology Center, Qingdao 266032, China; yanxin@cahec.cn (X.Y.); sunmingjun@cahec.cn (M.S.); huangmengkun@cahec.cn (M.H.); jinjihui@cahec.cn (J.J.); lijiaqi@cahec.cn (J.L.); sunxiangxiang@cahec.cn (X.S.); liumengda@cahec.cn (M.L.); zhanghaobo@cahec.cn (H.Z.); shaoweixing@cahec.cn (W.S.); sunshufang@cahec.cn (S.S.); 2 Key Laboratory of Animal Biosafety Risk Prevention and Control (South), Ministry of Agriculture and Rural Affairs, Qingdao 266032, China; 3 College of Agriculture, Yanbian University, Yanji 133000, China; 18943320397@163.com; 4 College of Veterinary Medicine, Qingdao Agricultural University, Qingdao 266109, China; 13225352723@163.com; 5 Key Laboratory of Ruminant Infectious Disease Prevention and Control (East), Ministry of Agriculture and Rural Affairs, Qingdao 266032, China

**Keywords:** brucellosis, chemiluminescent immunoassay, diagnosis, antibody testing

## Abstract

Brucellosis, a zoonotic infection caused by the intracellular pathogen *Brucella*, leads to chronic multi-organ damage. Currently, rapid, accurate, and sensitive diagnostic technologies are crucial for the prevention and control of brucellosis. This study describes the development of a chemiluminescent immunoassay (Bru-CLIA) for sheep and bovine brucellosis antibody detection, utilizing *Brucella abortus* strain A19 lipopolysaccharide-coated magnetic particles (LPS-MPs) as the serum antigen and acridinium ester-labeled recombinant streptococcal protein G (AE-SPG) for signal generation. After optimizing the assay’s parameters, the Bru-CLIA demonstrated a sensitivity of approximately 1 IU/mL and 2 IU/mL for detecting sheep and bovine brucellosis, respectively. No cross-reactivity was observed with sera from animals immunized with *Escherichia coli O157:H7*, *Mycobacterium tuberculosis*, *Vibrio cholerae*, *Legionella*, *Salmonella*, *Foot and Mouth Disease virus types O and A*, *Bovine viral diarrhea virus*, *Sheep contagious pleuropneumonia*, *Goat pox virus*, or *Peste des Petits Ruminants virus*, indicating strong specificity. The testing of 81 sheep serum samples and 96 bovine serum samples revealed that Bru-CLIA showed 87.65% and 93.75% concordance with the ID-VET commercial kits for sheep and bovine brucellosis detection, respectively. These results demonstrate that Bru-CLIA offers high specificity, sensitivity, repeatability, and reliability, making it a viable rapid diagnostic tool for the epidemiological surveillance of brucellosis.

## 1. Introduction

Brucellosis, a global zoonotic infection caused by the genus *Brucella*, leads to significant losses in animal husbandry, typically causing miscarriage and infertility in livestock [1]. In humans, it manifests with symptoms such as intermittent fever, excessive sweating, and arthritis, posing severe risks to health and life [2,3]. Effective control and prevention of brucellosis rely on timely and accurate diagnosis.

Currently, various methods are employed to detect brucellosis, including bacterial isolation, pathogen identification, and serological testing. Several pathogen detection techniques have been developed and validated, such as conventional PCR, real-time quantitative PCR (qPCR), droplet digital PCR (ddPCR), and recombinase-aided amplification (RAA) [4,5,6]. In contrast, serological tests are more commonly used for diagnosing suspected cases; surveillance; and epidemiological assessments [7]. Traditional serological tests, including the Rose Bengal Plate Test (RBPT), standard tube agglutination test (SAT), and complement fixation test (CFT), are still widely applied. While RBPT offers rapid screening through antigen–antibody agglutination, it suffers from a high false-positive rate [8]. SAT detects IgM antibodies, which are the first to appear after infection, but it is hampered by low specificity due to non-specific antigen–antibody interactions [9]. CFT, due to its technical complexity and challenges in standardization, is being increasingly replaced by more sensitive, rapid, and standardized techniques, such as enzyme-linked immunosorbent assay (ELISA) and fluorescence polarization assay (FPA) [10]. This study aims to develop a novel method for brucellosis antibody detection that supports high throughput and automation.

Chemiluminescence immunoassay (CLIA), a labeled immunoassay combining a chemiluminescent system with an immune reaction [11], is known for its high sensitivity and specificity. It has found widespread application in life science research, clinical medicine, food analysis, and environmental management, especially in human medicine [12,13,14]. Compared to agglutination tests, agar diffusion tests, and ELISA, CLIA stands out for its superior sensitivity, broad linear range, automation potential, and high throughput capability [15]. In veterinary medicine, CLIA has been successfully employed for diagnosing various animal diseases, such as African swine fever (ASF), foot-and-mouth disease (FMD), and Listeriosis. Yang et al. developed a CLIA using ASF virus (ASFV) protein p54 as the serum diagnostic antigen and an anti-p54 monoclonal antibody. This method exhibited a sensitivity of 1:128 and demonstrated a 100% concordance rate with the commercial kit in detecting 49 clinical samples [16]. Similarly, Liu et al. developed a CLIA for detecting antibodies against two recombinant epitope-based proteins located in 3A and 3B of FMD virus (FMDV). This CLIA showed an 88.1% concordance with the PrioCHECK FMDV NSP ELISA [17]. Additionally, Yue et al. developed a sandwich CLIA method for the detection of *Listeria monocytogenes*, employing a Cu/Co/Ni ternary nanoalloy as a catalyst. The method demonstrated a detection linear range from 2.0 × 10^2^ to 3.0 × 10^7^ CFU/mL and a minimum detection limit of 70 CFU/mL [18]. However, its use in brucellosis detection remains limited.

Lipopolysaccharide (LPS) is a critical factor in *Brucella*’s successful penetration into host cells and the evasion of the host’s immune system, playing a significant role in the diagnosis of sheep and bovine brucellosis and the study of its pathogenic mechanisms [19]. Commonly used diagnostic methods, such as ELISAs, frequently utilize LPS as the diagnostic antigen [20]. In this study, a novel CLIA-based method for detecting sheep and bovine brucellosis antibodies was developed, utilizing LPS-MPs as carriers and AE-SPG for signal generation. This assay, characterized by high sensitivity, automation capability, and broad linear range, offers a promising tool for the epidemiological surveillance of brucellosis in the “China Zoonosis Prevention and Control Plan 2022–2030” policy.

## 2. Materials and Methods

### 2.1. Material Sources

Bovine standard positive serum with a potency of 4000 IU/mL was obtained from the China Veterinary Drug Control Institute, while sheep standard positive serum with a potency of 400 IU/mL was provided by the China Animal Health and Epidemiology Center (CAHEC, Qingdao, China). A total of 33 sheep serum samples (13 brucellosis-positive and 20 brucellosis-negative) and 23 bovine serum samples (11 brucellosis-positive and 12 brucellosis-negative) were sourced from CAHEC (Qingdao, China). All brucellosis-positive sera were confirmed by RBPT and a commercial *Brucella* IgG ELISA kit (ID-VET, France), which utilizes *Brucella* LPS as the antigen. Negative serum samples were collected from a brucellosis-free area in China. Additionally, 81 sheep serum samples and 96 bovine serum samples were collected clinically. Positive sera for *Escherichia coli* O157:H7, *Mycobacterium tuberculosis*, *Vibrio cholerae*, *Legionella*, *Salmonella*, *Foot and mouth disease virus types O and A*, *Bovine viral diarrhea virus*, *Sheep contagious pleuropneumonia*, *Goat pox virus*, and *Peste des Petits Ruminants virus* were prepared in our laboratory. The Magnetic Particle Coating Kit and Acridine Lipid Labeling Kit were purchased from Boyuan Nuoxin Biotech (Beijing, China), while recombinant *Streptococcal* protein G (SPG) was sourced from Bersee Technology Limited (Beijing, China). Fetal Bovine Serum (FBS) was purchased from Gibco (Beijing, China) for system optimization and the dilution of bovine standard positive serum. The Automatic Chemiluminescence Immunoassay Analyzer Flash 200 was provided by Lvshiyuan Biotechnology Limited Company (Shenzhen, China).

### 2.2. LPS Extraction

*Brucella abortus* A19 was cultured to the exponential phase in Tryptic Soy Broth (TSB) and subsequently heat-inactivated at 80 °C for 2 h. Bacterial cells were collected by centrifugation, and LPS was extracted using a bacterial LPS extraction kit. Samples were loaded onto 12.5% polyacrylamide gels for SDS-PAGE, followed by silver staining as previously described [21]. The LPS was then freeze-dried and stored at −80 °C.

### 2.3. Conjugation of LPS to MPs

LPS was coupled to magnetic particles (MPs) with slight modifications to a previously described method [22]. In brief, 1 mg of beads was washed and resuspended in 900 μL binding buffer (0.1 M HEPES, pH 8.0). Then, 0.1 mg of LPS and 550 μL of a catalytic reagent solution were added, and the mixture was incubated for 18 h at 37 °C on a rotator. After incubation, the reaction was blocked with 100 μL of blocking reagent and further incubated for 2 h at 37 °C. The LPS-coated MPs were washed with washing buffer, resuspended in PBS, and stored at 4 °C.

### 2.4. Conjugation of AE to SPG

The SPG antibody was labeled with acridinium ester (AE) according to the following procedure. Briefly, 0.1 mg of SPG was dissolved in AE labeling buffer 4. After ultrafiltration, 100 μL of SPG at 1 mg/mL was obtained. Then, 4 μL of AE solution was added, and the reaction was carried out at 25 °C in the dark for 30 min. The reaction was blocked with 4 μL of blocking reagent and incubated for an additional 30 min at 25 °C in the dark with gentle shaking. Finally, AE labeling buffer 5, glycerol, and AE labeling protectant were added to the reaction solution to achieve a final AE-SPG concentration of 0.5 mg/mL.

### 2.5. Optimization of the Reaction System

To achieve the optimal conditions for Bru-CLIA, five critical parameters were optimized. Bovine standard positive serum was serially diluted with FBS, yielding concentrations of 500 IU/mL, 125 IU/mL, 31.25 IU/mL, 7.81 IU/mL, and 1.95 IU/mL. FBS was used as a negative control serum. The aforementioned six sera were used as a serum plate for the optimization of the reaction system. Other reaction conditions were tentatively set as follows: reaction diluent with 0.02 M PB, pH 7.4, magnetic bead concentration at 0.1 mg/mL (diluted with reaction diluent), acridinium ester concentration at 1:10,000 (diluted with reaction diluent), sample dilution at 5-fold (diluted with reaction diluent), and reaction procedure 1. The serum plate was subjected to triplicate testing, and the chemiluminescence values generated at each concentration point were recorded. The average values were calculated to determine the P/N ratio, and the group with the highest P/N ratio was selected as having the optimal reaction conditions.

1. Dilutions of LPS-MPs

LPS-MPs were diluted to concentrations of 0.2 mg/mL, 0.1 mg/mL, and 0.05 mg/mL. The other conditions were set as described in paragraph 2.5, section one. The serum plates were subjected to three repeated trials following the procedure, and the average values were calculated to determine the P/N ratios. The group with the highest P/N ratio was selected as the optimal LPS-MPs concentration.

2. Dilutions of AE-SPG

AE-SPG was diluted at ratios of 1:5000, 1:10,000, 1:15,000, and 1:20,000. Other conditions were kept as provisional, in line with the specifications detailed in paragraph 2.5, section one. Following the experimental protocol, the serum plates were tested three times with each dilution to establish reproducibility. The average values were then determined to calculate the P/N ratios. The group exhibiting the highest P/N ratio was identified as the optimal dilution factor for AE-SPG.

3. Dilutions of serum samples

Serum plates that had been diluted with reaction diluent at factors of 5, 10, 20, and 40 times were prepared. Other preliminary conditions were set according to those described in paragraph 2.5, section one. The experimental procedure was followed, with the serum plates subjected to three repeated trials. The average values were calculated to determine the P/N ratios. Based on these ratios, the optimal sample dilution factor was determined as the one yielding the highest P/N value.

4. Sample dilution buffers

Various types of reaction diluents were prepared, with their specific compositions as follows: buffer 1 (0.02 M PB, pH 7.4), buffer 2 (0.02 M Tris, pH 7.4), buffer 3 (0.02 M PB + 0.9% NaCl), buffer 4 (0.02 M Tris, pH 7.4 + 0.9% NaCl), buffer 5 (0.02 M PB, pH 7.4 + 0.9% NaCl + 0.1% Tween 20), buffer 6 (0.02 M PB, pH 7.4 + 0.9% NaCl + 0.2% Triton), and buffer 7 (1% casein sodium salt). Maintaining other conditions as provisionally described in paragraph 2.5, section one, three replicate experiments were conducted on the serum plates. The chemiluminescence values generated at each concentration point were recorded. Following the calculation of the average values, the P/N ratios were determined. The group with the highest P/N value was identified as the optimal reaction diluent.

5. Detection procedure

Four assay procedures were optimized: Procedure 1 (two steps) involved adding 50 μL of LPS-MPs, 5 μL of diluted serum sample, and 50 μL of dilution buffer to a test tube, followed by a 10-min incubation at 37 °C. After washing with TBST, 50 μL of AE-SPG, 100 μL of pre-excitation solution, and 100 μL of excitation solution were added, with a further 10-min incubation at 37 °C, after which RLU was measured. Procedure 2 (two steps) used 100 μL of LPS-MPs, 5 μL of diluted serum sample, and 100 μL of dilution buffer, followed by the same incubation steps. Procedure 3 (one step) involved combining 50 μL of LPS-MPs, 5 μL of diluted serum sample, 50 μL of dilution buffer, 50 μL of AE-SPG, 100 μL of pre-excitation solution, and 100 μL of excitation solution, followed by incubation for 10 min and RLU measurement. Procedure 4 (one step) involved adding 100 μL of LPS-MPs, 5 μL of diluted serum sample, 100 μL of dilution buffer, 100 μL of AE-SPG, 100 μL of pre-excitation solution, and 100 μL of excitation solution, followed by incubation for 10 min at 37 °C and RLU measurement.

### 2.6. Qualitative Analysis

To determine the cut-off value, diagnostic sensitivity, and specificity of Bru-CLIA, a total of 33 sheep serum samples and 23 bovine serum samples were tested. These parameters were calculated using receiver operating characteristic (ROC) curve analysis performed with GraphPad Prism version 10.0.0.

### 2.7. Analytical Sensitivity, Repeatability, and Cross-Reactivity

1. Sensitivity

The bovine standard positive serum was diluted to 500 IU/mL with FBS. Both the diluted bovine serum (500 IU/mL) and sheep standard positive serum (400 IU/mL) were serially diluted two-fold from 1:2 to 1:1024. The RLU values were simultaneously measured for each dilution in the Bru-CLIA. The highest dilution of serum that produced an RLU value exceeding the cut-off was considered the analytical sensitivity of the test.

2. Repeatability

Repeatability was assessed by calculating the intra- and inter-assay coefficients of variation (CVs). This was carried out using five sheep serum samples and five bovine serum samples with varying RLU values in three replicate experiments. The mean, standard deviation (SD), and percent coefficient of variation (CV) were computed.

3. Cross-reactivity

Cross-reactivity was assessed by testing positive sera against various pathogens, including *Escherichia coli O157:H7*, *Mycobacterium tuberculosis*, *Vibrio cholerae*, *Legionella*, *Salmonella*, *Foot and mouth disease virus types O and A*, *Bovine viral diarrhea virus*, *Sheep contagious pleuropneumonia*, *Goat pox virus*, and *Peste des Petits Ruminants virus*.

### 2.8. Comparison of Coincidence Rates

The concordance between Bru-CLIA and the ID-VET commercial ELISA kits was evaluated using 81 sheep serum samples and 96 bovine serum samples.

## 3. Results

### 3.1. Optimization of Working Condition

This study applied the developed Bru-CLIA to detect *Brucella* antibodies in sheep and bovine serum. To optimize its performance, key parameters were systematically studied and refined.

1. Optimization of LPS-MPs and AE-SPG concentration

**Figure 1:**
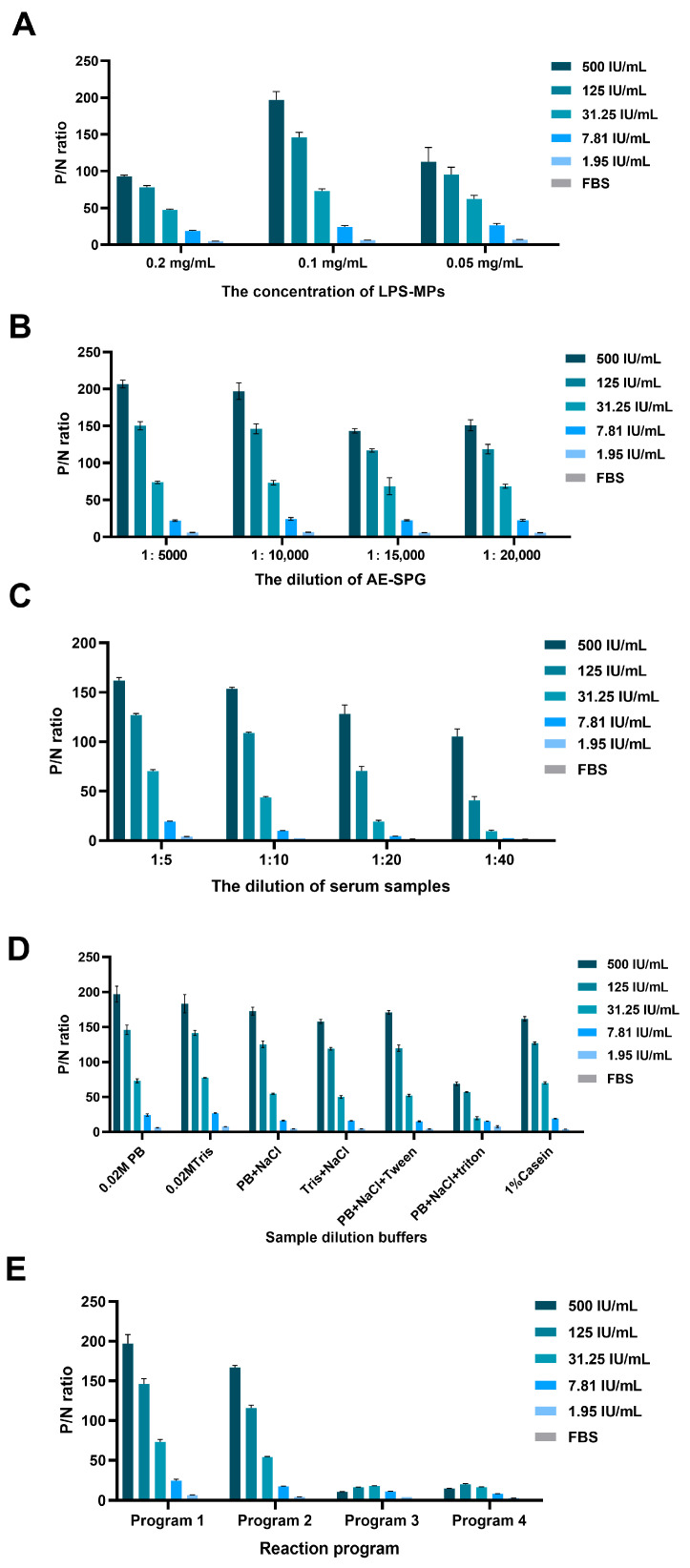
Establishment of Bru-CLIA. (**A**) The optimization of LPS-MPs at concentrations of 0.2 mg/mL, 0.1 mg/mL, and 0.05 mg/mL was performed in triplicate. (**B**) The optimization of AE-SPG at dilutions of 1:5000, 1:10,000, 1:15,000, and 1:20,000 was evaluated in triplicate. (**C**) The optimization of serum dilution at ratios of 1:5, 1:10, 1:20, and 1:40 was carried out in triplicate. (**D**) Optimization of sample dilution buffers: seven different diluents were used to dilute samples under the same conditions. (**E**) Detection procedure: both one-step and two-step incubation procedures for antigen–antibody reactions, including four programs, were evaluated.

The dilution ratios of LPS-MPs and AE-SPG were found to be critical factors influencing the sensitivity and specificity of the immunoassay. When LPS-MPs were diluted to 0.1 mg/mL, the P/N ratio reached its maximum value (Figure 1A). Similarly, a dilution of AE-SPG at 1:10,000 yielded the highest P/N ratio (Figure 1B). Therefore, 0.1 mg/mL of LPS-MPs and an AE-SPG dilution of 1:10,000 were selected for subsequent experiments.

2. Optimization of serum sample dilutions

For serum sample dilution, a ratio of 1:5 resulted in the highest P/N ratio (Figure 1C), so this dilution was chosen for further testing.

3. Optimization of sample diluent buffer

Seven sample dilution buffers were tested, including buffer 1 (0.02 M PB, pH 7.4), buffer 2 (0.02 M Tris, pH 7.4), buffer 3 (0.02 M PB + 0.9% NaCl), buffer 4 (0.02 M Tris, pH 7.4 + 0.9% NaCl), buffer 5 (0.02 M PB, pH 7.4 + 0.9% NaCl + 0.1% Tween 20), buffer 6 (0.02 M PB, pH 7.4 + 0.9% NaCl + 0.2% Triton), and buffer 7 (1% casein sodium salt). As shown in Figure 1D, serum diluted in 0.02 M PB, pH 7.4 produced the highest P/N ratio, making it the preferred buffer for subsequent tests.

4. Optimization of detection procedure

Regarding the detection procedure, both one-step and two-step assays were evaluated. Among the four procedures tested, two-step assay procedure 1 exhibited the highest P/N ratio (Figure 1E). Consequently, this procedure was selected for further testing.

### 3.2. Qualitative Analytical Performance

**Figure 2:**
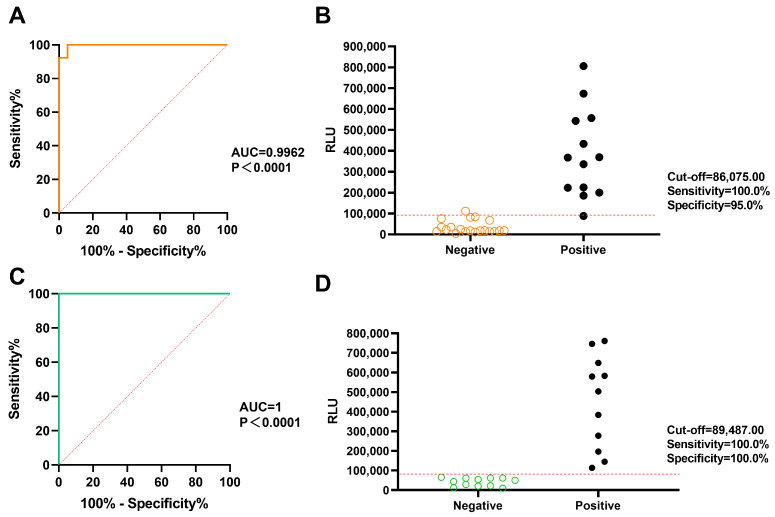
Determination of ROC and interactive dot diagrams of Bru-CLIA. (**A**,**B**) ROC curves and interactive dot diagrams for the detection of sheep serum samples. (**C**,**D**) ROC curves and interactive dot diagrams for the detection of bovine serum samples.

For qualitative analysis, a total of 33 sheep sera and 23 bovine sera were tested. The data were analyzed using GraphPad Prism version 10.0.0, and ROC curve analysis was conducted to calculate the cut-off value and generate an interactive scatter plot. In the detection of sheep brucellosis, the results indicated that at a cut-off value of 86,075, the diagnostic sensitivity was 100% and the diagnostic specificity was 95% (Figure 2A,B). For bovine brucellosis detection, a cut-off value of 89,487 resulted in diagnostic sensitivity and specificity of 100% (Figure 2C,D). The area under the curve (AUC) was 0.9962 for sheep brucellosis and 1.0 for bovine brucellosis, with *p* < 0.0001. An AUC value greater than 0.9 indicates excellent diagnostic accuracy. These results demonstrate the exceptional accuracy of the Bru-CLIA, particularly for bovine brucellosis detection.

### 3.3. Sensitivity, Cross-Reactivity, and Repeatability for Bru-CLIA

To assess the sensitivity of Bru-CLIA, serum samples with different dilution ratios were tested using the established Bru-CLIA method. For sheep and bovine brucellosis detection, the sensitivity of Bru-CLIA was 1:512 and 1:256, which corresponds to approximately 1 IU/mL and 2 IU/mL, respectively (Table 1).

The cross-reactivity of the Bru-CLIA was evaluated using both positive and negative brucellosis sera, as well as positive sera for *Escherichia coli* O157:H7, *Mycobacterium tuberculosis*, *Vibrio cholerae*, *Legionella*, *Salmonella*, *Foot and mouth disease virus types O and A*, *Bovine viral diarrhea virus*, *Goat pox virus*, and *Small ruminant plague virus*. The results demonstrated that the developed Bru-CLIA did not exhibit cross-reactivity with these sera (Table 2), indicating its strong specificity

Ten brucellosis sera, comprising 5 sheep and 5 bovine serum samples, were tested. The CVs for intra- and inter-batch repeatability ranged from 0.9% to 4.2% and 0.3% to 8.1%, respectively (Table 3). All results were below 10%, indicating the strong stability of the Bru-CLIA. 

### 3.4. Comparison of Coincidence Test

To assess the suitability of the detection method developed in this study for clinical brucellosis antibody detection, a concordance test was conducted between Bru-CLIA and commercial kits. As shown in Table 4, Bru-CLIA demonstrated 87.65% and 93.75% concordance with the ID-VET ELISA commercial kits for detecting sheep and bovine brucellosis, respectively. The Kappa consistency coefficient for sheep clinical samples is 0.732, which indicates good agreement. The Kappa consistency coefficient for cattle clinical samples is 0.855, which indicates almost perfect agreement (<0.20 poor, 0.21–0.40 fair, 0.41–0.60 moderate, 0.61–0.80 good, and 0.81–1.00 perfect). These results suggest that Bru-CLIA is a promising diagnostic method for the clinical diagnosis of sheep and bovine brucellosis.

## 4. Discussion

Serological tests play a pivotal role in diagnosing human and animal brucellosis. While pathogen isolation and nucleic acid testing are essential, serodiagnostic methods such as RBT and ELISA are prioritized in many endemic regions due to their ease of implementation in clinical settings with limited resources [23,24]. RBT is commonly used as an initial screening test for brucellosis, offering high specificity but relatively low sensitivity. ELISA, while providing both good sensitivity and specificity, is expensive and requires well-trained technicians [10]. CLIA utilizes chemiluminescent reactions in immunochemical assays. Based on light emission principles, it is classified into direct chemiluminescence (CLIA), chemiluminescent enzyme immunoassay (CLEIA), and electrochemiluminescence immunoassay (ECLIA) [11]. CLIA offers several advantages, including full automation, reduced human intervention, and more reliable results, making it suitable for widespread use at the grassroots level. Additionally, CLIA is extensively used in human medical diagnostics and is increasingly replacing ELISA in China [25,26]. Due to its high sensitivity, good specificity, rapid turnaround time, and ease of automation, this method has been widely applied in the detection of diseases such as *Treponema pallidum*, *breast cancer*, *hepatitis E virus*, and *COVID-19* [27,28,29,30]. Currently, it also holds significant potential for application in veterinary diagnostics, for example, in the detection of ASFV and FMDV, owing to its high sensitivity, good specificity, rapid turnaround time, and ease of automation. However, research on the application of CLIA in brucellosis diagnosis remains limited.

In this study, Bru-CLIA was established based on LPS-MPs and AE-SPG. The method demonstrated pertinent specificity, sensitivity, and rapidity in detecting sheep and bovine brucellosis antibodies (Table 1, Table 2 and Table 3). Notably, Bru-CLIA replaces the traditional 96-well plate format with MPs. MPs offer a larger specific surface area, strong magnetism, and excellent biological compatibility, and they enable automated detection, which reduces manual handling and improves both throughput and consistency [31]. The sensitivity of Bru-CLIA was twice that of the ID-VET ELISA commercial kits. Another key factor in evaluating diagnostic methods is detection time, which directly influences the efficiency of the assay [32]. Bru-CLIA utilizes AE as a signal probe, a typical flash-type chemiluminescent substance [33]. Signal recording with AE is much faster than traditional markers such as alkaline phosphatase (AP) or horseradish peroxidase (HRP). Thus, Bru-CLIA completed assays within 40 min, offering a rapid and user-friendly solution for high-throughput testing. Furthermore, no cross-reactions were observed with sera positive for *Escherichia coli* O157:H7, *Mycobacterium tuberculosis*, *Vibrio cholerae*, *Legionella*, *Salmonella*, *foot-and-mouth disease virus types O and A*, *Bovine viral diarrhea virus*, *Goat pox virus*, or *Small ruminant plague virus*.

This study is the first to evaluate and analyze Bru-CLIA for the clinical diagnosis of brucellosis. For detecting sheep and bovine brucellosis, the cut-off fluorescence values were 86,075 and 89,487, respectively, with AUC values of 0.9962 and 1.0, demonstrating excellent diagnostic accuracy. The concordance rates between Bru-CLIA and the ID-VET ELISA commercial kits were 87.65% and 93.75%, respectively. Bru-CLIA showed greater sensitivity and faster results than ELISA, providing significant technological support for epidemiological surveillance, immune efficacy evaluation, and even the quantitative analysis of standard serum.

## Figures and Tables

**Table 1:** Analytical sensitivity test of Bru-CLIA.

Sample	Dilution Ratios	Bru-CLIA *^a^*		Dilution Ratios	Bru-CLIA *^b^*	
		RLU	Results		RLU	Results
Positive control sera	1:2	814,436	+	1:2	922,104	+
	1:4	759,971	+	1:4	845,213	+
	1:8	674,499	+	1:8	729,834	+
	1:16	567,784	+	1:16	564,003	+
	1:32	459,471	+	1:32	441,203	+
	1:64	352,746	+	1:64	271,532	+
	1:128	246,878	+	1:128	154,402	+
	1:256	168,844	+	1:256	89,634	+
	1:512	108,461	+	1:512	51,530	−
	1:1024	76,730	−	1:1024	25,818	−

*^a^* Sheep sera. *^b^* Bovine sera. RLU: Relative Luminescence Unit.

**Table 2:** Analytical specificity test of Bru-CLIA.

Pathogens	RLU	Results
*Escherichia coli* O157:H7	3364	−
*Mycobacterium tuberculosis*	2894	−
*Vibrio cholerae*	3652	−
*Legionella*	3180	−
*Salmonella*	3556	−
*Foot and mouth disease virus types O and A*	2426	−
*Bovine viral diarrhea virus*	3065	−
*Goat pox virus*	1980	−
*Small ruminant plague virus*	2978	−
*Brucella*-negative serum	2515	−
*Brucella*-positive serum	270,045	+

**Table 3:** Analytical repeatability test of Bru-CLIA.

Serum Number	Intra-Batch Coefficient of Variation		Coefficient of Variation Between BATCHES	
	Mean ± SD	CV (%)	Mean ± SD	CV (%)
1 *^a^*	562,542 ± 5936	1.1	567,451 ± 9916	1.8
2 *^a^*	466,142 ± 6566	1.4	458,471 ± 1060	0.3
3 *^a^*	349,255 ± 12,118	3.5	351,746 ± 9758	2.8
4 *^a^*	243,968 ± 7600	3.1	246,544 ± 4891	2.0
5 *^a^*	166,790 ± 1453	0.9	168,844 ± 3538	2.1
6 *^b^*	729,834 ± 6381	0.9	875,931 ± 53,582	6.1
7 *^b^*	564,003 ± 8427	1.5	656,737 ± 45,050	6.9
8 *^b^*	441,203 ± 18,290	4.2	352,904 ± 6763	1.9
9 *^b^*	271,532 ± 7782	2.9	115,738 ± 9328	8.1
10 *^b^*	154,402 ± 5820	3.8	29,086 ± 2098	7.2

*^a^* Sheep sera. *^b^* Bovine sera.

**Table 4:** Comparison of coincidence rates between Bru-CLIA and ELISA.

Methods	Samples	Bru-CLIA			
		PS.N	NS.N	Total	Coincidence Rate
ID-VET ELISA *^a^*	PS.N	24	6	30	80.00%
	NS.N	4	47	51	92.16%
	Total	28	53	81	87.65%
Kappa: 0.732					
ID-VET ELISA *^b^*	PS.N	27	2	29	93.10%
	NS.N	4	63	67	94.03%
	Total	31	65	96	93.75%
Kappa: 0.855					

*^a^* Sheep sera. *^b^* Bovine sera. ID-VET ELISA: ID-VET Commercial iELISA Kit. PS.N: Positive Sample Number. NS.N: Negative Sample Number.

## Data Availability

The original contributions presented in the study are included in the article. Further inquiries can be directed to the corresponding authors.
